# When David Surrenders the Sling: The Misalignment of Science and Strategy in Biotech-Pharma Partnerships

**DOI:** 10.7759/cureus.95608

**Published:** 2025-10-28

**Authors:** Shaheen E Lakhan

**Affiliations:** 1 Department of Neurology, Western University of Health Sciences, Pomona, USA; 2 Department of Neurology, A.T. Still University School of Osteopathic Medicine in Arizona, Mesa, USA; 3 Department of Neurology, Morehouse School of Medicine, Atlanta, USA; 4 Department of Bioscience, Global Neuroscience Initiative Foundation, Miami, USA

**Keywords:** biotechnology, commercialization, digital therapeutics, innovation policy, partnerships, pharmaceutical industry, scientific integrity

## Abstract

Partnerships between small biotechnology companies and large pharmaceutical corporations are essential to translating early discoveries into global therapies. Yet many of these alliances falter, not because of scientific failure, but because of structural, cultural, and ethical misalignment. What begins as a collaboration of complementary strengths often devolves into an unequal relationship, where the agility and innovation of the biotech are absorbed by the slower machinery of pharma. The result is stalled progress, diluted science, and lost opportunities for patients.

This editorial examines the growing divide between small, platform-driven biotechs and large pharmaceutical partners, particularly as the frontier of medicine expands into digital, data-driven, and multimodal therapeutics. It explores how governance asymmetry, conflicting time horizons, and the commandeering of scientific authorship erode trust and value. Drawing on firsthand experience across multiple global collaborations, I argue that the future of medicine demands a new model of partnership, one that protects scientific sovereignty while enabling strategic alignment. True collaboration must evolve from transactional handoffs to the shared creation of enduring platforms that unite biology, behavior, and technology.

## Editorial

Introduction: the promise and the paradox

In theory, the relationship between a small biotech and a large pharmaceutical company is one of natural synergy. The biotech brings creativity, speed, and risk tolerance; the pharma partner contributes resources, scale, and regulatory experience. Together, they should accelerate innovation and deliver transformative therapies to patients. In practice, the story often unfolds quite differently.

Across the industry, partnerships that begin with mutual admiration frequently end in frustration or quiet dissolution [1]. Misaligned incentives, asymmetric power structures, and incompatible cultures conspire to turn collaboration into confrontation [2]. Small biotechs discover that the very qualities that made them attractive, agility, bold science, and singular focus, are gradually constrained by bureaucratic processes, conservative decision-making, and commercial imperatives.

In my own experience leading and negotiating partnerships across continents, I have seen promising programs stall not because of the science but because of the system built to manage it. When collaboration becomes control, innovation loses momentum.

Structural asymmetry: governance and incentives

At the foundation of most failed alliances lies a structural imbalance. The biotech’s objective is survival through rapid value creation. The pharma’s objective is risk mitigation and portfolio diversification. Each sees time differently: for the biotech, every month matters; for the pharma, every quarter is a rounding error in a decade-long plan.

Joint steering committees, designed to harmonize decision-making, often become arenas of procedural paralysis. Veto rights, layered approvals, and risk-averse governance erode the nimbleness that made the biotech valuable in the first place. Even well-intentioned oversight can slow clinical execution to a crawl.

Milestone structures amplify this tension. Payments are tied to regulatory events that align with pharma’s accounting rhythms rather than the biotech’s operational needs. Delays in data transfer or protocol amendments can freeze a biotech’s cash flow, forcing it to seek bridge financing for work already completed. In many cases, the smaller company bears disproportionate responsibility for early development, while the larger partner reserves control of commercialization decisions, effectively decoupling effort from reward.

The asymmetry extends beyond money to information. Data-sharing clauses are often restrictive, and access to raw data may be delayed or mediated through corporate channels under standard collaboration frameworks such as joint development or data-use agreements. These contracts typically include confidentiality provisions, publication-review periods, and data-access governance clauses that require mutual consent before disclosure, mechanisms intended to protect intellectual property and competitive positioning. Yet in practice, they often result in asymmetric transparency, where the smaller partner must share data upward while receiving little reciprocal visibility. The flow of knowledge becomes one-directional: from biotech to pharma.

Cultural and operational divides

Even when contracts are sound, cultures clash. Startups thrive on improvisation; large companies thrive on process. The biotech’s success depends on flexibility and rapid iteration, while the pharma’s value lies in predictability and compliance. These differences can create a kind of organizational immune response, the larger entity treats the smaller one as a foreign body, surrounding it with layers of regulation until its vitality is subdued.

This cultural divide is particularly visible in emerging areas such as digital therapeutics, artificial intelligence, and platform-based R&D. Traditional pharma operates within a molecule-centric paradigm. Biotech and tech-enabled companies, by contrast, think in systems: code, algorithms, and data loops that evolve continuously. When a digital or data-driven biotech partners with pharma, the latter often tries to impose a clinical-trial framework designed for static molecules onto a dynamic platform. The result is a mismatch of expectations and timelines.

Operationally, this means endless debates about software version control, quality management, and labeling conventions for products that defy traditional categories. What is a “drug label” for a therapeutic algorithm that learns over time? Who owns the patient-generated data? How is safety monitored in a system that updates itself? These questions are rarely answered in advance, and without shared philosophical alignment, they become friction points that stall progress.

The commandeering of science

Perhaps the most insidious misalignment, however, is not structural or cultural but intellectual. Once a collaboration is in place, control over the scientific narrative often shifts decisively to the larger partner [1]. The biotech’s scientists, who conceived the platform, designed the trials, and generated the data, find themselves marginalized in authorship, publication planning, and conference presentations.

I have seen this pattern repeatedly: manuscripts delayed for months pending internal review, abstracts rewritten to align with corporate branding, and data reframed to fit a broader franchise strategy. In the name of consistency and compliance, pharma frequently commandeers the storytelling of the science itself.

Authorship disputes are common, particularly when publication committees are dominated by the partner with greater regulatory infrastructure. Even when credits are shared, the order often implies a hierarchy that belies the true origin of the work. Scientists who spent years building the intellectual foundation may see their contributions condensed into an acknowledgment.

This is not merely a matter of ego; it affects transparency and progress [3]. When smaller companies cannot freely publish mechanistic findings, exploratory analyses, or patient-reported outcomes, the broader scientific community loses valuable insight. In some cases, publication embargoes extend until after a commercial launch, rendering the findings less relevant and reducing the opportunity for peer validation.

Scientific commandeering undermines morale, distorts incentives, and erodes the trust upon which partnerships depend. It also creates a chilling effect on innovation: biotechs may become hesitant to share early discoveries, fearing they will lose control of the narrative once a deal is signed.

Balancing science and business

The heart of the problem lies in an enduring misconception that science and business are separate domains to be negotiated, rather than integrated pursuits to be aligned. Too often, partnership negotiations focus on intellectual property and financial milestones while neglecting governance of scientific integrity.

Biotechs must learn to view scientific communication as a strategic asset, not a postscript [1]. The ability to publish, present, and interpret one’s own data is essential to sustaining credibility, attracting talent, and maintaining investor confidence. It is also a moral obligation to patients and peers.

In every collaboration agreement, there should be a clear publication charter, detailing authorship order, review timelines, and rights to publish non-confidential results [4]. Independent scientific steering committees can ensure that data interpretation is guided by evidence, not by brand positioning. Transparency clauses should protect the biotech’s ability to publish mechanistic or exploratory findings even when commercial decisions are pending.

For pharma, respecting scientific sovereignty is not charity but enlightened self-interest. When a partner’s science is visible and validated, the shared asset gains legitimacy and the alliance earns credibility. Trust is not built through nondisclosure agreements; it is built through shared truth.

When business overrules biology

In many alliances, business strategy quietly supersedes biological rationale. Promising programs are deprioritized not because they lack efficacy or safety but because they do not fit a partner’s therapeutic franchise or market narrative. What begins as a scientific collaboration becomes a portfolio optimization exercise.

I have witnessed entire therapeutic areas abandoned due to shifting corporate priorities. A digital therapeutic designed for schizophrenia may be shelved when a new oncology asset promises higher margins. A novel mechanism of neuroplasticity modulation may languish because it lacks a convenient comparator for phase-three benchmarking. These decisions may make business sense in isolation, but they undermine the cumulative progress of medical science.

The irony is that both parties lose. The biotech forfeits momentum and morale; the pharma forfeits innovation and goodwill. Patients, of course, lose most of all.

Balancing business imperatives with biological truth requires leadership that understands both domains. Scientists must learn to speak the language of markets, and executives must respect the grammar of science. The best alliances are led by bilingual leaders, those who can translate between the logic of molecules and the logic of money without corrupting either.

The rise of platform and digital biotechs

The challenge of alignment is magnified in the new generation of platform and digital biotechs. Unlike traditional asset-based companies that develop a single molecule or device, these firms build engines capable of producing multiple products across indications. Their value lies not only in any one therapy but in the architecture that enables scalable innovation.

Pharma, however, often approaches these partnerships with a legacy mindset, seeking to in-license a discrete asset rather than to co-develop a platform. This misalignment leads to transactional deals that undervalue the underlying technology and overconstrain its evolution.

For example, when a digital therapeutic platform designed to treat depression, anxiety, and insomnia enters partnership discussions, the pharma partner may insist on indication-specific exclusivity. What seems prudent risk management to pharma becomes existential constraint for the biotech, effectively freezing its broader pipeline. Similarly, platform improvements that span multiple programs may be slowed by change-control processes meant for static medical devices.

In these cases, the partnership fails not because the science is weak but because the framework cannot accommodate a living, learning technology. Platform biotechs operate on iteration; pharma operates on validation. Bridging that gap requires not compromise but reinvention of the collaboration model itself.

Toward a new model of partnership

If the current model is broken, what should replace it? The answer is not fewer partnerships but better ones, rooted in transparency, parity, and shared purpose.

First, partnerships must be structured for alignment, not absorption. The goal should be co-creation of value rather than transfer of ownership. This means preserving the biotech’s autonomy in scientific decision-making and ensuring that governance committees reflect true balance, not symbolic representation.

Second, incentives must be outcome-based. Milestones should reward not only regulatory achievements but demonstrable patient benefit, real-world performance, and scientific contribution. In digital and platform collaborations, metrics such as engagement, adherence, and data quality should complement traditional clinical endpoints.

Third, data transparency must be contractual. Both parties should have symmetrical access to de-identified clinical and mechanistic data. Shared dashboards and data rooms can reduce friction and foster joint learning.

Fourth, scientific authorship should be codified as a right, not a privilege. Joint publication committees should include equal representation, with timelines that respect scientific relevance. A default posture of openness, publish unless prohibited, not silence unless approved, would accelerate collective progress.

Finally, cultural integration must be intentional. Embedding liaison scientists, conducting reciprocal secondments, and aligning compliance training can help both sides appreciate each other’s constraints. The best alliances I have seen were those where pharma executives spent time inside the biotech’s lab, and biotech scientists participated in the pharma’s global medical meetings. Proximity breeds empathy; empathy sustains partnership.

From transaction to transformation

The relationship between biotech and pharma is evolving from a transactional exchange of molecules to a transformational collaboration of minds. As medicine becomes increasingly digital, personalized, and data-driven, no single entity can succeed alone. The next generation of therapies will emerge at the intersection of biology, behavior, and technology, a space that demands both the imagination of startups and the discipline of industry.

For this partnership to thrive, however, both sides must relinquish old habits. Pharma must learn to share control without fearing chaos; biotech must learn to scale without losing soul. Each must recognize that the ultimate stakeholder is not the shareholder but the patient.

In Puerto Rico, where I founded Boricua Bio, the ethos of innovation is inseparable from community, the idea that progress must serve people, not just profit. I believe that spirit should guide the next era of biotech-pharma collaboration. Science with corazón.

Conclusion: reclaiming scientific sovereignty

When David surrenders the sling, Goliath may win the battle but lose the war of innovation. The small biotech’s strength lies not in size but in focus, creativity, and conviction. The large pharma’s strength lies in reach, rigor, and resources. When properly aligned, these forces can move medicine forward faster than either could alone.

But alignment cannot be achieved through contracts alone. It requires a shared commitment to truth over optics, collaboration over control, and science over spin. The future of medicine will be built not on who owns the data but on who honors it.

The world no longer needs more mergers; it needs more meaning. In every partnership, the question should not be “Who leads?” but “Who learns?” The answer to that question will determine not only the success of the alliance but also the trajectory of modern therapeutics.

If biotech and pharma can find that equilibrium, between autonomy and alignment, between science and business, then perhaps the next generation of partnerships will not end in misalignment but in discovery. And that, after all, is the purpose of science.
